# I can’t wait! An investigation into time processing in cocaine use disorder

**DOI:** 10.1192/j.eurpsy.2022.344

**Published:** 2022-09-01

**Authors:** T. Burke, K. Ersche

**Affiliations:** University of Cambridge, Department Of Psychiatry, Cambridge, United Kingdom

**Keywords:** Impulsivity, Cocaine use disorder, time reproduction

## Abstract

**Introduction:**

Almost all definitions of impulsivity include the notion of distorted time perception such as impaired awareness of the future or premature responses. Preclinical evidence suggests that stimulant drugs speed up the internal clock, making time pass faster than it actually is. However, stimulant-addicted humans, who are drug-abstinent seem to over-estimate long time intervals.

**Objectives:**

The present study aims to investigate time processing in actively using patients with cocaine use disorder (CUD). We hypothesise that active cocaine use will be associated with an under-estimation of long time intervals.

**Methods:**

We recruited 48 men with a chronic history of cocaine use, meeting the DSM-5 criteria for CUD, and 42 healthy men without a history of substance use disorders. All participants completed a time reproduction task in which they were presented four times with six different time durations and were subsequently asked to reproduce them by pressing the space bar for the same time duration of the target interval they had just seen. Participants also completed the Barratt Impulsiveness Scale (BIS-11).

**Results:**

Overall precision in time reproduction was significantly reduced in CUD patients (F_6,81_=3.97,p=0.002), which was particularly evident for longer time delays. CUD patients’ estimated-to-target-duration ratios were marginally shorter for the 11000ms (F_1,86_=3.1,p=0.084) and significantly shorter for the 18000ms and 24000ms time intervals (both p<0.05). Time reproduction performance correlated with self-reported attentional impulsivity on the BIS-11 in both CUD patients and healthy controls (all p<0.05).

**Conclusions:**

Consistent with preclinical work, the inner clock of humans with regular cocaine use seems to be accelerated.

**Disclosure:**

No significant relationships.

